# Discharge time from healthcare facilities after birth in Tanzania : a secondary analysis of demographic and health surveys from 2015 to 16 and 2022

**DOI:** 10.1186/s12913-026-14035-x

**Published:** 2026-01-14

**Authors:** Aline Semaan, Lise Apers, Amani Kikula, Thomas van den Akker, Andrea Barnabas Pembe, Lenka Beňová, Natasha Housseine

**Affiliations:** 1 Department of Public Health, Institute of Tropical Medicine, Antwerp, Belgium; 2 Athena Institute, Vrije Universiteit Amsterdam, Amsterdam, the Netherlands; 3 Global Health Institute, Faculty of Medicine and Health Sciences, University of Antwerp, Antwerp, Belgium; 4 Department of Obstetrics and Gynaecology, Muhimbili University of Health and Allied Sciences, Dar es Salaam, Tanzania; 5 Department of Obstetrics and Gynaecology, Leiden University Medical Centre, Leiden, the Netherlands; 6 Global Health Section, Department of Public Health, University of Copenhagen, Copenhagen, Denmark; 7 Medical College East Africa, Aga Khan University, Dar es Salaam, Tanzania

**Keywords:** Maternal, Newborn, Healthcare facility, Childbirth, Postpartum, Postnatal, Household survey, Tanzania, Vaginal birth, Caesarean section, Demographic and health survey

## Abstract

**Background:**

Care during the immediate postpartum period is critical for women and newborn’s health. Following facility-based birth, it is recommended to monitor the health of women and newborns in the facility. In Tanzania, a third of women who give birth in a health facility report not receiving a health check during their stay. Duration of stay and factors influencing them are not well documented in Tanzania. We describe postpartum length-of-stay at two time-points, 2015/2016 and 2022 in Tanzania, and explore factors associated with early discharge.

**Methods:**

We analysed secondary data from two Demographic and Health Surveys: *n* = 3,582 women from 2015 to 16 and *n* = 4,618 from 2022 who had a livebirth in a health facility during the three years preceding the survey. Early discharge was defined as discharge < 24 h after vaginal birth and < 72 h after a caesarean birth. We describe the percentage of early discharge at both DHS time-points and explore factors associated with it using 2022 data with multivariable logistic regression, separately by mode of birth.

**Results:**

Almost half of women who had a vaginal birth were discharged early in 2015-16, compared to 30.3% in 2022. Early discharge increased among women who had a caesarean section from 17.2% in 2015-16 to 25.2% in 2022. The odds of early discharge were higher in Zanzibar compared to Eastern zone, and factors associated with early discharge included level of facility, frequency of antenatal care visits, maternal education and newborn underweight status.

**Conclusions:**

Despite progress in reducing early postpartum discharge after vaginal births, it continued to affect a third of women giving birth in Tanzania in 2022. Disparities between regions and facility levels suggest potential inconsistencies in the application of postpartum guidelines, warranting the need to determine and address the underlying causes, standardise practice and ensure equitable access to quality postpartum care.

**Supplementary Information:**

The online version contains supplementary material available at 10.1186/s12913-026-14035-x.

## Introduction

Maternal mortality remains high globally with an estimated 260,000 maternal deaths in 2023, whereby most of these deaths occur in low- and middle-income countries (LMIC) [1]. The global maternal mortality ratio (MMR) was estimated at 197 maternal deaths per 100,000 livebirths, whereas in Africa the MMR was 410 deaths per 100,000 livebirths in 2023 [1]. Common causes of maternal deaths include haemorrhage, hypertensive disorders and sepsis, and most maternal deaths occur in the postpartum period [2]. Additionally, around 1.9 million intrapartum stillbirths occurred in 2021, and 2.3 million newborns died in 2022, 1 million of these deaths occurred during the first hour after birth [3, 4]. This highlights the need for early postnatal care (PNC) during the first week after birth, for closely monitoring the mother’s and newborn’s health, and detecting and managing complications timely [5, 6].

The latest World Health Organization (WHO) recommendations advise a minimum 24 h stay in a healthcare facility after vaginal birth, enabling continuous care and monitoring for women and newborns [5]. The WHO underscores the importance of delivering these recommendations within an appropriate model of PNC, tailored to fit the needs, resources and local contexts of countries or regions [5]. A model of PNC refers to the organisation of care provision elements such as length-of-stay after facility-based childbirth, and the provider, location, frequency, timing, duration of postnatal checks, including care provided before discharge. Evidence to inform the organisation of PNC models remains limited, as highlighted by two Cochrane reviews showing gaps in research on postpartum length-of-stay and the frequency, timing, and duration of home visits [7–9]. Additionally, a scoping review on discharge preparedness guidelines shows potential gaps between evidence and policies globally [6]. As a result, PNC models and the recommended length-of-stay after childbirth vary globally, with many LMIC offering stays too short to provide PNC of adequate quality [10, 11]. Several factors determine the timing of discharge, and these are complex and at multiple levels of the socio-ecological framework, and vary globally [10, 12]. This underscores the need to examine postpartum length-of-stay in healthcare facilities and its determinants.

In Tanzania, the MMR was estimated at 104 per 100,000 livebirths, and the neonatal mortality rate was 14 per 1,000 livebirths in 2022 [13]. National policies have paid closer attention to expanding the availability of facility-based childbirth care, increasing the percentage of births in facilities with skilled birth attendants and improving the availability of management for obstetric complications [14, 15]. As a result, progress has been made over the last decades, leading to 85% of livebirths being attended by a skilled healthcare professional [13]. The National Postpartum Care guidelines of the Tanzanian Ministry of Health (MoH) recommends that women and newborns stay 24 h at the healthcare facility after birth [15]. This period allows monitoring vital signs and bleeding, early initiation of exclusive breastfeeding and counselling parents on hygiene, family planning, danger sign identification and umbilical cord care, amongst others [16]. However, evidence on postpartum length-of-stay and determinants of time of discharge is lacking in Tanzania. This study aims to describe postpartum length-of-stay in healthcare facilities in Tanzania and to explore factor associated with early discharge. Specifically, the study first describes how postpartum length-of-stay and the percentage of early discharge changed between two time-points: 2015/2016 and 2022 among women who gave birth in healthcare facilities in Tanzania. Second, the study determines factors (socio-demographic, economic, facility-level, women’s needs and obstetric history and newborn characteristics) associated with early discharge in the most recent timepoint (2022).

## Methods

### Study setting

Tanzania, located in Eastern Africa, is a LMIC comprising 31 administrative regions [13]. The healthcare system follows a pyramidal structure with primary healthcare (including community care) at the base and the National Referral Hospital at the apex [17]. Most facilities in Tanzania provide childbirth services, and one in five offers comprehensive emergency obstetric and newborn care (CEmONC), which encompasses services such as blood transfusions and caesarean secions [17]. Most livebirths (81%) occur in a healthcare facility, with 75% of them occurring in governmental facilities [13]. Adherence to the recommended number and timing of PNC check-ups is low in the country and challenges to quality of care have been repeatedly described [18–21]. One third of women reported receiving a postnatal check-up within four hours of birth, and 44% reported not having received any postnatal checks [13]. Less than 10% of newborns were reported to receive the first postnatal check-up within the first hour of birth [13]. Notable regional variability is observed within the country both in terms of maternal and newborn PNC, with coverage being higher in urban compared to rural areas [13, 22]. Qualitative evidence suggests that healthcare providers often acknowledge having insufficient time, resources and space which prevents them from providing the quality of postnatal education they aspire to deliver, and requires them to reduce the time that women spend in the healthcare facility postpartum [23–25].

### Study design, data and population

**Fig. 1:**
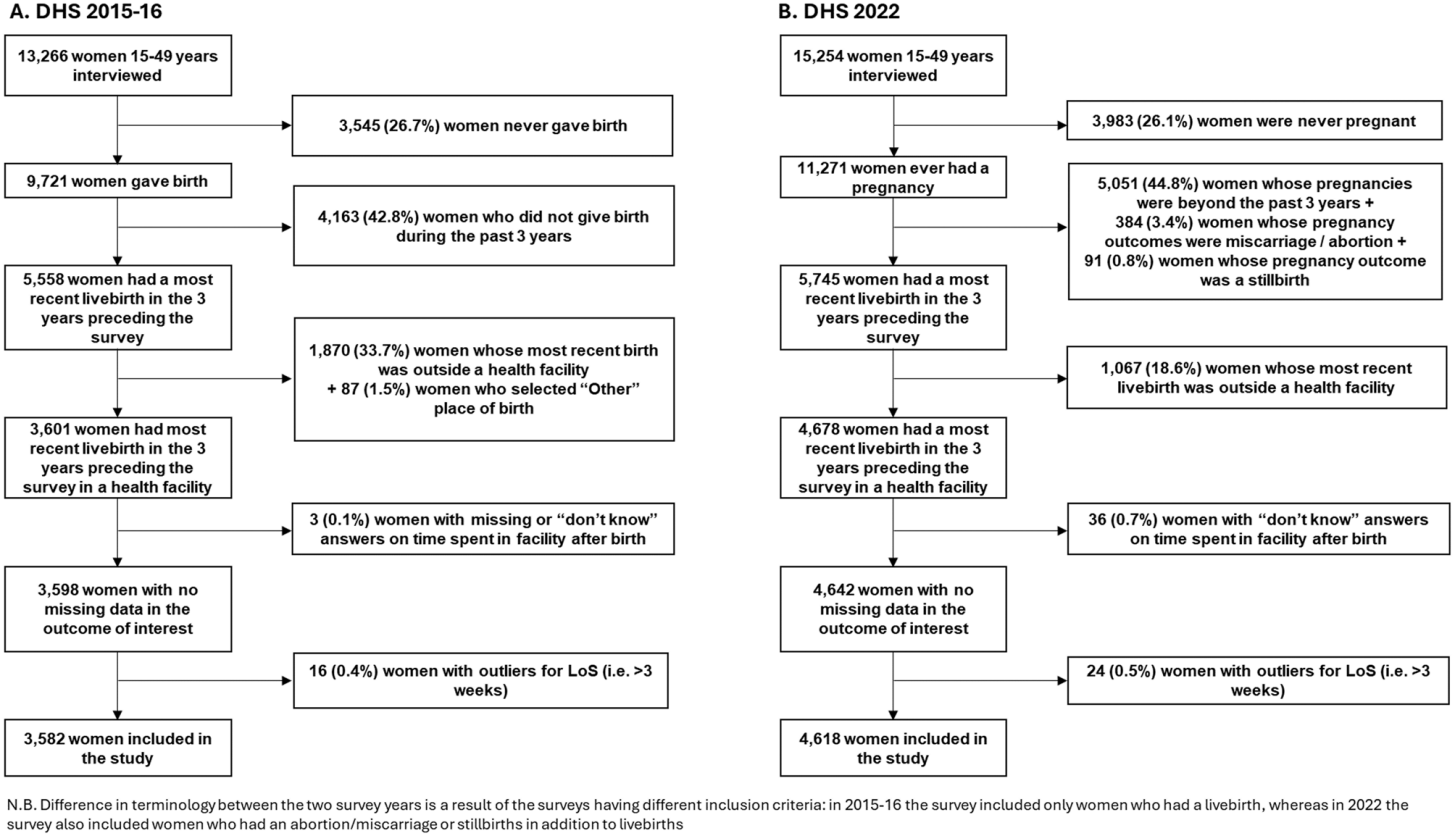
Flowchart of inclusion criteria and final sample size of women who participated in the Tanzania DHS (unweighted percentages shown)

This is a quantitative cross-sectional study using secondary data from two Tanzanian Demographic and Health Surveys (DHS): 2015–2016 and 2022. DHS are nationally representative household surveys of key health and population indicators, which used to take place every 5–6 years per country to monitor change in demographic and health indicators over time [26]. In this study, we used data from the two most recently surveys. Figure 1 shows the flowchart of inclusion/exclusion criteria by survey year. The DHS survey interviews women aged 15–49 at the time of data collection. In the 2015/16 survey, the questions to women were about the most recent birth that occurred during the five years preceding the survey. In the 2022 survey, the recall period changed to births occurring in the three years preceding the survey. Thus to ensure that the samples in both surveys are comparable, we included only births that took place in the three years preceding the survey. We excluded births that took place outside a healthcare facility since our research question is only relevant to births in healthcare facilities. Births occurring before the three year period preceding the survey, and stillbirths, were excluded because they were not captured in both surveys. Women who had missing data or outlier values on the outcome variable (postpartum length-of-stay) were excluded from the analysis.

### Outcome and independent variables

The outcome of interest is postpartum length-of-stay, representing the time that women spent in a healthcare facility after their most recent livebirth and before discharge. The DHS questionnaire includes the question: “*How long after (NAME) was delivered did you stay there?*”, where the answers are continuous and expressed in hours, days or weeks. We unified the unit of time to hours accounting for the bias introduced by the unit of time according to Campbell et al. [10], and excluded outliers with length-of-stay > 3 weeks (Fig. 1, *n* = 16 in 2015-16 and *n* = 24 in 2022). The continuous variable was categorised as early discharge depending on women’s reported mode of birth. Based on the Tanzanian MoH’s postpartum care guideline and WHO PNC recommendations, early discharge was defined as discharge before 24 h for women who had a vaginal birth, and before 72 h for those who gave birth by caesarean section [5, 15].

Independent variables, described in Additional File 1, Table S1, were selected based on a thorough literature review and following the conceptual framework of determinants of postpartum length-of-stay developed by Campbell et al. [10]. We included factors theoretically linked to postpartum length-of-stay and available in the DHS datasets, and spanning four levels: (1) socio-demographic and economic factors (zone, residence, union and cohabiting status, maternal age at index childbirth, education, household wealth index); (2) Facility characteristics and perception of care quality (facility level and ownership, whether women shared bed during stay, spent time without mattress on the floor, were denied care, delayed to leave facility because could not pay or if they had access to a toilet; 3) women’s obstetric history (mode of birth, parity at index birth, antenatal care frequency during index pregnancy, multiple births, previous terminated pregnancy); and 4) newborn characteristics (sex, underweight status at birth, newborn survival/time of death).

### Analysis

We described sample characteristics by survey year with frequencies and percentages with 95% confidence intervals. Length-of-stay was described separately by mode of birth (vaginal and caesarean section) and survey year, both as a continuous variable in hours (median) and categorized as early discharge vs. not. Results were displayed in tables and visualised in graphs. Associations between early discharge and independent variables were explored with logistic regression using data from 2022 DHS survey, separately by mode of birth (Additional file 1 Figure S1). Factors with p-value < 0.2 were included in multivariable logistic regression models. The variable on newborn survival was excluded from the regression model among women who had a cesarean section because of small sample size in sub-categories. We used Stata SE v.16, and statistical significance level was set at p-value < 0.05. All analyses adjusted for the stratified sampling strategy and sampling weights of the DHS.

### Missing values

Three (0.1%) women in 2015-16 and 36 (0.7%) in 2022 had missing values for length-of-stay and were excluded (Fig. 1). Their characteristics are described in Additional file 1 Table S2.

The independent variable ‘newborn weight’ had 213 missing values in 2015-16 and 334 in 2022. In the 2022 survey, there were missing values for the variables on self-reported quality and respectful care. Two of these variables were eligible to be included in the multivariable logistic regression model on early discharge. We conducted a sensitivity analysis by excluding the variables from the models (Additional file 1 Table S3), which showed no impact on direction, strength, or significance of associations.

## Results

### Sample characteristics

Table 1 describes characteristics of 3,582 and 4,618 women who had their most recent livebirth in a healthcare facility in the three years preceding the 2015–16 and 2022 DHS surveys. In both periods, the most common age group at birth was 20–24 years. 51.6% in 2015–16 and 38.9% in 2022 reported giving birth in a hospital. The percentage of women giving birth in government-owned facilities was 80.5% in 2015–16 and 91.6% in 2022. Births by caesarean section were reported by 10.1% of women in 2015-16 and 13.1% in 2022.

**Table 1:** Characteristics of women (15–49) who had their most recent livebirth in a healthcare facility in the three years preceding the DHS surveys 2015-16 and 2022

	Characteristics	DHS 2015-16 (*n* = 3582)		DHS 2022 (*n* = 4618)	
		n (%)†	95%CI	n (%)†	95%CI
Socio-demographic and economic factors	**Zone**				
	Western	285 (9.8%)	[8.1;11.8]	416 (10.4%)	[8.9;12.0]
	Northern	291 (10.3%)	[8.6;12.2]	372 (9.5%)	[8.4;10.8]
	Central	334 (10.7%)	[9.1;12.5]	375 (9.5%)	[8.2;10.9]
	Southern highlands	347 (7.2%)	[6.2;8.3]	408 (6.6%)	[5.9;7.3]
	Southern	195 (5.3%)	[4.3;6.6]	219 (5.1%)	[3.9;6.4]
	South west highlands	384 (10.1%)	[8.3;12.4]	622 (10.1%)	[8.9;11.5]
	Lake	771 (23.7%)	[21.6;25.9]	999 (30.4%)	[27.4;33.6]
	Eastern	452 (20.1%)	[17.8;22.5]	497 (15.4%)	[13.4;17.6]
	Zanzibar	523 (2.8%)	[2.4;3.2]	710 (3.0%)	[2.6;3.4]
	**Residence**				
	Rural	2401 (62.0%)	[59.2;64.7]	3109 (67.5%)	[62.6;71.9]
	Urban	1181 (38.0%)	[35.3;40.8]	1509 (32.5%)	[28.1;37.4]
	**Union and cohabiting status at time of survey**				
	Not in union/not living with a partner	888 (26.2%)	[24.3;28.1]	1260 (28.0%)	[26.4;29.6]
	Living with a partner	2694 (73.8%)	[71.9;75.7]	3358 (72.0%)	[70.4;73.6]
	**Maternal age at index childbirth**				
	13–19 years	429 (13.3%)	[11.9;14.9]	419 (10.0%)	[8.9;11.2]
	20–24 years	990 (28.3%)	[26.6;30.0]	1200 (26.9%)	[25.3;28.5]
	25–29 years	838 (23.3%)	[21.6;25.1]	1210 (26.3%)	[24.7;27.9]
	30–34 years	653 (18.2%)	[16.6;19.8]	845 (17.6%)	[16.3;18.9]
	35–49 years	672 (16.9%)	[15.6;18.3]	944 (19.2%)	[17.9;20.5]
	**Highest completed education level**				
	No formal education	481 (13.1%)	[11.6;14.8]	727 (16.1%)	[14.4;17.8]
	Primary education	2135 (64.0%)	[61.7;66.3]	2381 (56.3%)	[54.4;58.2]
	Secondary or higher	966 (22.9%)	[20.9;24.9]	1510 (27.6%)	[25.6;29.8]
	**Household wealth index**				
	Poorest	618 (17.3%)	[15.1;19.7]	796 (17.5%)	[15.4;19.8]
	Poorer	648 (20.4%)	[18.4;22.5]	862 (19.7%)	[17.9;21.6]
	Middle	654 (19.5%)	[17.7;21.4]	928 (20.7%)	[19.1;22.5]
	Richer	715 (21.5%)	[19.6;23.6]	991 (21.3%)	[19.6;23.2]
	Richest	947 (21.3%)	[18.9;23.7]	1041 (20.8%)	[18.0;23.9]
Facility characteristics and perception of care quality	**Facility level**				
	Dispensary/clinic	880 (25.3%)	[22.9;27.8]	1425 (33.0%)	[30.4;35.8]
	Health center	849 (23.1%)	[20.8;25.5]	1248 (28.1%)	[26.0;30.3]
	Hospital	1853 (51.6%)	[48.8;54.4]	1945 (38.9%)	[36.2;41.6]
	**Facility Ownership**				
	Governmental	2962 (80.5%)	[77.9;82.8]	4256 (91.6%)	[90.2;92.9]
	Non-governmental	620 (19.5%)	[17.2;22.1]	362 (8.4%)	[7.1;9.8]
	**Day of birth**				
	Weekend	1009 (28.5%)	[26.7;30.4]	1182 (24.8%)	[23.4;26.3]
	Weekday	2573 (71.5%)	[69.6;73.3]	3436 (75.2%)	[73.7;76.6]
	Shared bed during stay*	n/a		558 (11.3%)	[9.9;12.7]
	Spent time without mattress on the floor*	n/a		119 (3.0%)	[2.3;3.7]
	Was denied care because could not pay*	n/a		150 (3.9%)	[3.2;4.8]
	Was delayed in leaving facility because could not pay*	n/a		141 (3.8%)	[3.0;4.6]
	Access to toilet*	n/a		3887 (84.2%)	[82.7;85.6]
Women’s needs and obstetric history	**Mode of birth**				
	Vaginal	3258 (89.9%)	[88.4;91.3]	3994 (86.9%)	[85.4;88.3]
	Caesarean section	324 (10.1%)	[8.7;11.6]	624 (13.1%)	[11.7;14.6]
	**Parity at index birth**				
	Primiparous	1075 (31.9%)	[30.1;33.9]	1133 (24.9%)	[23.4;26.5]
	Multiparous 2–4	1681 (47.9%)	[45.7;50.0]	2400 (53.5%)	[51.5;55.4]
	Grand multiparous 5+	826 (20.2%)	[18.6;21.8]	1085 (21.6%)	[20.0;23.3]
	**Number of ANC visits during index pregnancy**				
	None	28 (0.9%)	[0.5;1.4]	447 (9.3%)	[8.2;10.6]
	1–3 visits	1596 (43.1%)	[40.9;45.3]	976 (21.0%)	[19.3;22.7]
	4 or more visits	1958 (56.0%)	[53.8;58.3]	3195 (69.7%)	[67.7;71.6]
	**Multiple birth**	67 (1.7%)	[1.2;2.2]	75 (1.8%)	[1.3;2.5]
	**Ever had a terminated pregnancy**	609 (16.4%)	[15.0;17.8]	627 (12.6%)	[11.5;13.8]
Newborn characteristics	**Newborn sex**				
	Boy	1851 (53.1%)	[51.2;55.0]	2362 (51.7%)	[49.9;53.4]
	Girl	1731 (46.9%)	[44.9;48.8]	2256 (48.3%)	[46.6;50.0]
	**Newborn underweight status at birth** ^**β**^				
	Underweight (< 2500 g)	216 (6.1%)	[5.2;7.2]	311 (7.3%)	[6.3;8.2]
	Not underweight (≥ 2500 g)	3153 (93.9%)	[92.8;94.8]	3973 (92.7%)	[91.8;93.6]
	**Newborn survival**				
	Survived until survey	3462 (96.6%)	[95.9;97.2]	4518 (97.7%)	[96.9;98.2]
	Died on/before discharge	47 (1.3%)	[0.9;1.8]	46 (1.1%)	[0.7;1.7]
	Died after discharge	73 (2.1%)	[1.6;2.7]	54 (1.2%)	[0.9;1.7]

Abbreviations: Antenatal care (ANC); Confidence interval (CI); Demographic and Health Survey (DHS). †Percentages are adjusted for the sampling weights of the DHS data. *Missing values: shared bed *n* = 1; Spent time on floor *n* = 3; denied care *n* = 2; delayed to leave *n* = 13; access to toilet *n* = 8

^β^*n* = 213 missing values for newborn’s weight at birth in 2015-16; *n* = 334 missing values in 2022

n/a – not available on the 2015-6 DHS

### Postpartum length-of-stay and early discharge

**Fig. 2:**
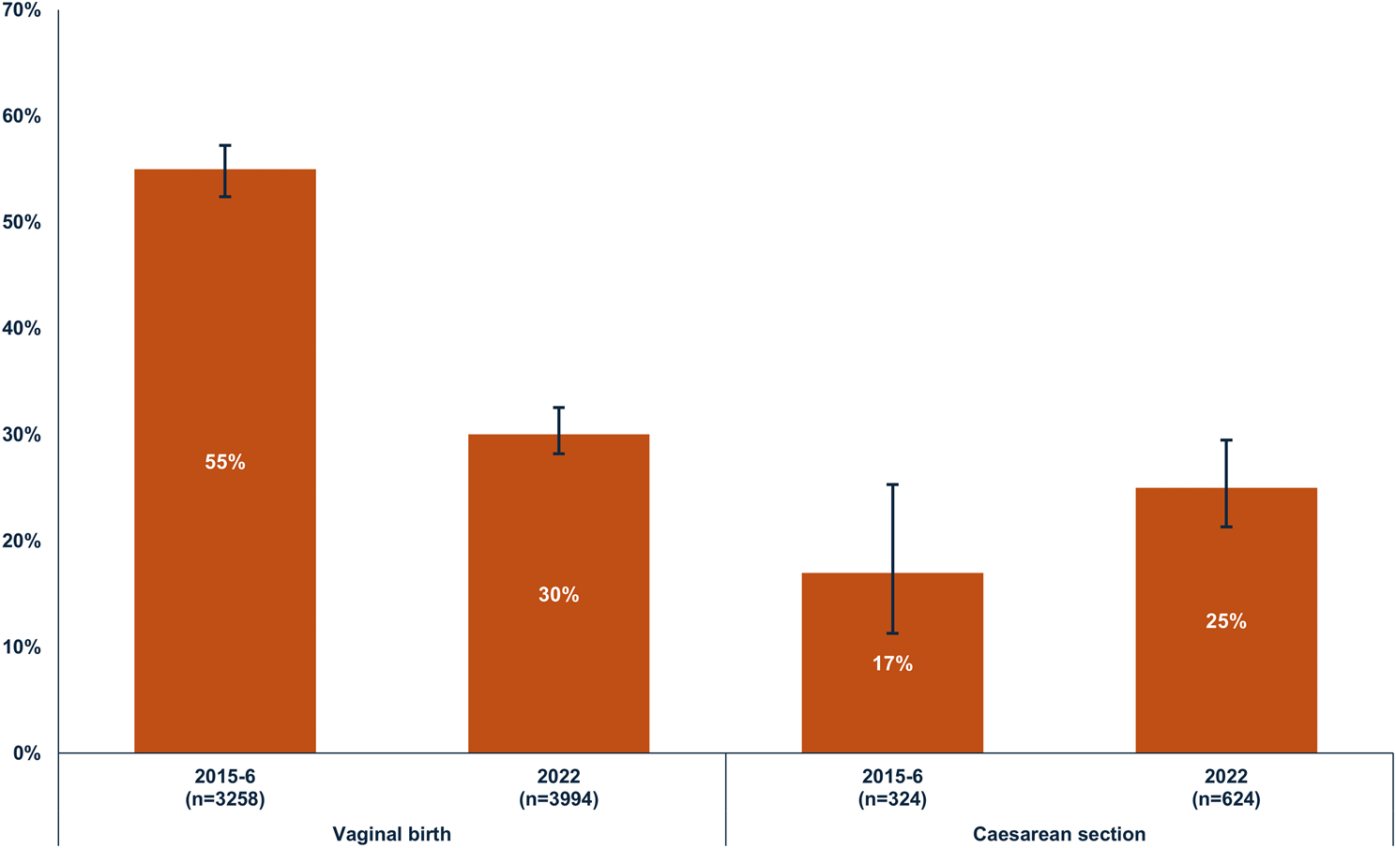
Percentage of early discharge* among women who had their most recent livebirth in the three years preceding the survey, by mode of birth and DHS survey year. *Early discharge is defined as leaving the healthcare facility in less than 24 h after a vaginal birth or less than 72 h after birth by caesarean section

Among women who had a vaginal birth, the median time spent in the facility increased from 16 h [IQR = 6–36] in 2015-16 to 24 h [IQR = 12–36] in 2022. Among women who gave birth by caesarean section, the median time spent in the facility was 84 h in both survey years. Additional file 1 Table S4 describes postpartum length-of-stay and early discharge in 2015-16 and 2022. Figure 2 shows the percentage of women discharged early from healthcare facilities by mode of birth and survey year. Among women who had a vaginal birth, 54.8% [95% CI = 52.4;57.2] were discharged early in 2015-16 and 30.3% [95% CI = 28.2; 32.5] in 2022. The percentage of early discharge among women who had a caesarean section was 17.2% [95% CI = 11.3;25.3] in 2015-16 and 25.2% [95% CI = 21.3;29.5] in 2022.

### Factors associated with early discharge – DHS 2022

The results of bivariate and multivariable logistic regressions determining factors associated with early discharge are in Table 2 for women who had a vaginal birth and Table 3 for women who had a caesarean section.

#### Vaginal birth

Fourteen factors associated with early discharge with a p-value ≤ 0.2 in bivariate regression were included in the multivariable model. Upon adjusting, three factors were significantly associated with early discharge among women who had a vaginal birth: zone, facility level and number of ANC checks. The odds of early discharge were higher in Zanzibar (aOR = 6.3) and South west highlands (aOR = 1.8) compared to Eastern zone; while the odds of early discharge were lower in Southern Highlands (aOR = 0.5) compared to Eastern zone. The odds of early discharge among women who gave birth in a health centre were 1.5 times higher compared to those who gave birth in a hospital. Similarly, the odds of early discharge among those who gave birth in a dispensary/clinic were 3.7 times higher than hospital-based births. Women who had no ANC checks during the index pregnancy had lower odds of early discharge compared to those who had at least four checks (Table 2).

#### Caesarean section

For women who had a caesarean section, eight factors were included in the multivariable regression model. Upon adjusting, three factors were significantly associated with early discharge: education level, level of facility of birth, and newborn underweight status at birth. Women who had no formal education had 0.3 times lower odds of early discharge compared to those who completed primary education. Women who had caesarean section in a lower-level facility (health centre/dispensary/clinic) had 3.3 times the odds of early discharge compared to those who had it in a hospital. Women whose newborns were underweight had five times lower odds of early discharge compared to those whose babies weighed ≥ 2500 g at birth (Table 3).

**Table 2:** Bivariate and multivariable logistic regression of factors and early discharge among women who had their most recent *vaginal birth* in a healthcare facility in the three years preceding the DHS 2022 (total *n* = 3994*)

	Characteristics	cOR [95%CI]	*p*-value	aOR [95%CI]	*p*-value
Socio-demographic and economic factors	**Zone**†				
	Western	0.8 [0.5;1.2]	0.232	0.6 [0.4;1.0]	0.063
	Northern	1.0 [0.6;1.4]	0.804	1.2 [07;1.9]	0.500
	Central	0.8 [0.6;1.2]	0.320	0.7 [0.5;1.1]	0.154
	Southern highlands	0.5 [0.3;0.8]	0.003	0.5 [0.3;0.8]	0.006
	Southern	1.4 [0.8;2.4]	0.237	0.9 [0.6;1.5]	0.717
	South west highlands	2.3 [1.6;3.2]	< 0.001	1.8 [1.2;2.8]	0.004
	Lake	0.7 [0.5;1.0]	0.042	0.8 [0.5;1.1]	0.136
	Eastern	ref		ref	
	Zanzibar	3.7 [2.7;5.0]	< 0.001	6.3 [4.0;9.8]	< 0.001
	**Residence**				
	Rural	ref		ref	
	Urban	0.7 [0.6;0.9]	0.012	1.0 [0.7;1.3]	0.920
	**Union and cohabiting status at time of survey**				
	Not in union/not living with a partner	ref		ref	
	Living with a partner	1.2 [0.9;1.5]	0.082	1.2 [0.9;1.4]	0.193
	**Maternal age at index childbirth**				
	13–19 years	0.9 [0.7;1.3]	0.718	1.0 [0.7;1.4]	0.851
	20–24 years	ref		ref	
	25–29 years	1.1 [0.8;1.4]	0.475	1.0 [0.8;1.4]	0.827
	30–34 years	1.2 [0.9;1.5]	0.134	1.2 [0.8;1.6]	0.391
	35–49 years	1.3 [1.0;1.6]	0.045	1.1 [0.8;1.7]	0.503
	**Highest completed education level**				
	No formal education	1.2 [0.9;1.5]	0.147	1.0 [0.8;1.3]	0.772
	Primary education	ref		ref	
	Secondary or higher	0.8 [0.7;1.0]	0.149	0.9 [0.8;1.7]	0.503
	**Household wealth index**				
	Poorest	1.1 [0.9;1.5]	0.329	1.0 [0.7;1.4]	0.959
	Poorer	0.8 [0.6;1.1]	0.120	0.8 [0.6;1.1]	0.151
	Middle	ref		ref	
	Richer	0.9 [0.7;1.1]	0.247	0.9 [0.7;1.2]	0.526
	Richest	0.8 [0.6;1.1]	0.119	0.9 [0.6;1.3]	0.414
Facility characteristics and perception of care quality	**Facility level**†				
	Dispensary/clinic	3.2 [2.4;4.2]	< 0.001	3.7 [2.7;5.1]	< 0.001
	Health center	1.2 [0.9;1.6]	0.118	1.5 [1.1;2.0]	0.012
	Hospital	ref		ref	
	**Facility ownership**				
	Governmental	ref		ref	
	Non-governmental	0.6 [0.4;0.9]	0.016	0.9 [0.6;1.4]	0.588
	**Day of birth**				
	Weekend	ref			
	Weekday	1.0 [0.9;1.2]	0.720		
	**Shared bed during stay**				
	No	ref			
	Yes	1.1 [0.8;1.4]	0.680		
	**Spent time without a mattress on the floor**				
	No	ref			
	Yes	1.1 [0.6;1.9]	0.797		
	**Was denied care because could not pay**				
	No	ref		ref	
	Yes	0.7 [0.4;1.2]	0.158	0.7 [0.8;1.6]	0.463
	**Was delayed in leaving facility because could not pay**				
	No	ref		ref	
	Yes	0.5 [0.3;0.9]	0.016	0.6 [0.3;1.2]	0.122
	**Access to toilet**				
	No	ref			
	Yes	1.0 [0.8;1.3]	0.826		
Women’s needs and obstetric history	**Parity at index birth**				
	Primiparous	ref		ref	
	Multiparous 2–4	1.3 [1.0;1.6]	0.034	1.0 [0.7;1.3]	0.796
	Grand multiparous 5+	1.6 [1.3;1.9]	< 0.001	1.1 [0.8;1.6]	0.559
	**Number of ANC visits during index pregnancy**†				
	None	0.7 [0.5;1.0]	0.079	0.6 [0.4;0.9]	0.023
	1–3 visits	1.1 [0.9;1.3]	0.536	1.0 [0.8; 1.2]	0.710
	4 or more visits	ref		ref	
	**Multiple birth**				
	No	ref			
	Yes	0.6 [0.3;1.4]	0.224		
	**Ever had a terminated pregnancy**				
	No	ref			
	Yes	1.0 [0.8;1.3]	0.904		
Newborn characteristics	**Newborn sex**				
	Boy	ref			
	Girl	0.9 [0.8;1.2]	0.817		
	**Newborn underweight status at birth**				
	Not underweight ≥ 2500 g	ref		ref	
	Underweight < 2500 g	0.7 [0.5;0.9]	0.032	0.8 [0.5;1.1]	0.114
	**Newborn survival**				
	Survived until survey	ref		ref	
	Died on/before discharge	0.5 [0.2;1.3]	0.154	0.4 [0.1;1.3]	0.117
	Died after discharge	0.5 [0.2;1.2]	0.135	0.7 [0.3;1.7]	0.392

Abbreviations: Antenatal care (ANC); Confidence interval (CI); Crude odds ratio (cOR); Adjusted odds ratio (aOR). *n = 323 missing values between the variables ‘delayed to leave the facility’ and ‘newborn weight’. †Factors with significant association with early discharge after vaginal birth in the multivariable model

**Table 3:** Bivariate and multivariable logistic regression of factors and early discharge among women who had their most recent livebirth by *Cesarean section* in a healthcare facility within three years of DHS 2022 (*n* = 624)

	Characteristics	cOR [95%CI]	*p*-value	aOR [95%CI]	*p*-value
Socio-demographic and economic factors	**Zone**†				
	Western	0.7 [0.2;2.0]	0.508	0.8 [0.2;2.7]	0.704
	Northern	0.8 [0.4;1.5]	0.426	0.8 [0.4;1.8]	0.590
	Central	1.0 [0.5;2.2]	0.902	1.5 [0.6;4.0]	0.363
	Southern highlands	0.5 [0.3;1.0]	0.050	0.6 [0.3;1.3]	0.171
	Southern	0.7 [0.2;2.0]	0.490	0.9 [0.3;2.8]	0.841
	South west highlands	0.7 [0.3;1.5]	0.385	0.8 [0.4;2.0]	0.683
	Lake	1.0 [0.4;2.4]	0.966	1.3 [0.5;3.3]	0.514
	Eastern	ref		ref	
	Zanzibar	1.4 [0.5;3.7]	0.503	1.9 [0.7;5.1]	0.230
	**Residence**				
	Rural	ref		ref	
	Urban	1.4 [0.9;2.2]	0.097	1.5 [0.9;2.5]	0.099
	**Union and cohabiting status at time of survey**				
	Not in union/not living with a partner	ref			
	Living with a partner	0.9 [0.6;1.5]	0.807		
	**Maternal age at index childbirth**				
	13–19 years	1.4 [0.5;3.5]	0.521		
	20–24 years	ref			
	25–29 years	1.2 [0.6;2.1]	0.600		
	30–34 years	1.6 [0.8;3.1]	0.179		
	35–49 years	0.8 [0.4;1.6]	0.474		
	**Highest completed education level reached**†				
	No formal education	0.3 [0.1; 0.8]	0.022	0.3 [0.1;0.9]	0.031
	Primary education	ref		ref	
	Secondary or higher	1.1 [0.7; 1.6]	0.689	1.0 [0.6;1.7]	0.975
	**Household wealth index**				
	Poorest	0.4 [0.1;1.3]	0.124	0.6 [0.2;1.6]	0.289
	Poorer	1.1 [0.5;2.2]	0.822	1.3 [0.6;2.8]	0.552
	Middle	ref		Ref	
	Richer	1.4 [0.7;2.7]	0.301	1.5 [0.8;2.9]	0.259
	Richest	1.3 [0.7;2.5]	0.400	1.5 [0.7;3.2]	0.251
Facility characteristics and perception of care quality	**Facility level**†				
	Lower level facilities - Health center/dispensary/clinic	2.3 [1.4;3.8]	0.001	3.3 [2.0;5.5]	< 0.001
	Hospital	ref		ref	
	**Facility Ownership**				
	Governmental	ref		ref	
	Non-governmental	0.6 [0.3;1.2]	0.181	0.8 [0.4;1.5]	0.424
	**Day of birth**				
	Weekend	ref			
	Weekday	0.8 [0.5;1.4]	0.507		
	**Shared bed during stay**				
	No	ref			
	Yes	1.4 [0.7;2.7]	0.388		
	**Spent time without a mattress on the floor**				
	No	ref		ref	
	Yes	2.5 [0.8;8.1]	0.117	1.7 [0.5;5.7]	0.410
	**Was denied care because could not pay**				
	No	ref			
	Yes	1.1 [0.4;2.9]	0.835		
	**Was delayed in leaving facility because could not pay**				
	No	ref			
	Yes	1.4 [0.5;3.6]	0.488		
	**Access to toilet**				
	No	ref			
	Yes	0.7 [0.4;1.3]	0.290		
Women’s needs and obstetric history	**Parity at index birth**				
	Primiparous	ref			
	Multiparous 2–4	1.1 [0.7;1.7]	0.732		
	Multiparous 5+	0.7 [0.4;1.4]	0.327		
	**Number of ANC visits during index pregnancy**				
	None	1.2 [0.5;2.6]	0.730		
	1–3 visits	0.9 [0.5;1.6]	0.658		
	4 or more visits	ref			
	**Multiple birth**				
	No	ref			
	Yes	0.9 [0.3;3.2]	0.966		
	**Ever had a terminated pregnancy**				
	No	ref			
	Yes	0.7 [0.4;1.4]	0.316		
Newborn characteristics	**Newborn sex**				
	Boy	ref			
	Girl	1.0 [0.7;1.5]	0.888		
	**Newborn underweight status at birth**†				
	Not underweight ≥ 2500 g	ref		ref	
	Underweight < 2500 g	0.2 [0.1;0.7]	0.013	0.2 [0.1;0.7]	0.014

Abbreviations: Antenatal care (ANC); Confidence interval (CI); Crude odds ratio (cOR); Adjusted odds ratio (aOR).*n = 23 missing values between the variables ‘delayed to leave the facility’ and ‘newborn weight’. †Factors with significant association with early discharge after cesarean birth in the multivariable model

## Discussion

This paper describes change between two time periods in postpartum length-of-stay and early discharge from healthcare facilities in Tanzania, and factors associated with it among women who had a livebirth in the three years preceding DHS 2022. A third of women who gave birth in a health facility were discharged in < 24 h after a vaginal birth and < 72 h after a caesarean section in 2022. Between 2015/16 and 2022, the percentage of early discharge among women who had a vaginal birth (< 24 h) declined, and the opposite was observed among postpartum women discharged < 72 h after a caesarean birth. Significant regional differences in early postpartum discharge exist, and factors associated with early discharge included level of facility of birth, frequency of ANC visits, maternal education and newborn underweight status.

Over the examined period, the percentage of women discharged < 24 h after a vaginal birth nearly halved. The MoH’s recommendation of 24 h stays did not change during this period [15, 16]. The observed change is possibly linked to incremental efforts applied over time to implement those recommendations. Traditionally, women who give birth vaginally are perceived by care providers as having low-risk and possibly discharged earlier than 24 h. The observed trend could signal a shift from these perceptions and a targeted effort to monitor women and newborns for longer hours postpartum. It is also possible that the upgrading of lower-level facilities to provide surgery care [27] increased bed space availability, particularly in higher level healthcare facilities, potentially reducing the need to discharge women early to create space for others [25].

The percentage of early discharge among women who gave birth via caesarean section (< 72 h) increased between the two periods (from 17% in 2015-16 to 25% in 2022). Globally, postpartum length-of-stay after caesarean section is declining over time [28]; the ERAS program [29] which aims to reduce recovery time post-surgery has been partially implemented in Tanzania, however it is unlikely that it would have an impact at the national level considering that it is not fully institutionalised. During the COVID-19 pandemic, postpartum length-of-stay declined at a faster rate, as women preferred leaving the facility quickly and providers aimed to decongest facilities and reduce risk of infection transmission [30–32]. The data included in this study covers the period of the pandemic – a period when reductions in postpartum length-of-stay were among adaptations implemented in the Tanzanian national referral hospital [32]. Another hypothesis is that the increase in the caesarean section rate (from 7% to 10%) [13] could mean that a larger percentage of all caesarean sections were among women without major complications (e.g. scheduled caesarean section), suggesting that more women who had a caesarean section could recover and leave the health facility earlier. Another possibility is that the demand for post-operative beds increased with the increasing caesarean section rate beyond bed availability and women were discharged earlier to make space for others [25]. Last, it is possible that fewer women were being detained for unpaid fees [33–35] in 2022 and were able to leave the facility earlier, although more research is needed to explore these hypotheses.

Despite the reduction in percentage of early postpartum discharge over time, it was documented among a third of women included in the study. This proportion of early discharge following vaginal birth in Tanzania is comparable to estimates in Cameroon (29.7%) [36], India (22.3%) [37], and Eswatini (22%); and higher than Malawi (10%) [38]. Early discharge < 72 h after a caesarean birth was higher in Tanzania compared to Cameroon (15.1%) and India (12.8%) [36, 37]. This suggests challenges related to prioritisation of immediate PNC, guideline implementation, space availability, staffing, among other health-system issues hindering provision of quality care in the immediate postpartum period [39]. The results of the multivariable analysis show that early discharge is determined by health-system level factors. Women who gave birth in lower-level facilities might be less likely to suffer from complications compared to women who give birth in higher-level facilities, therefore might be considered by providers that they can recover easier and be discharged before the recommended length-of-stay. This higher odds of early discharge in lower-level facilities could also be a result of having less resources, including infrastructure, space availability, and staff, compared to hospitals. Additionally, this signals a potential difference in adherence to guidelines between the levels of the healthcare system. In a context where lower-level facilities are being upgraded to provide CEmONC [27], efforts should be made to ensure the same quality of care is available across all levels of the healthcare system. The results also highlight geographic differences with early discharge being higher in Zanzibar compared to other geographic zones. This could be a result of lack of space availability in Zanzibar due to congestion of hospitals leading to discharging women as soon as safe for them to free-up beds. Another explanation could be that there is inconsistent implementation of recommendations between zones, suggesting potential inequalities in the provision and quality of immediate postpartum care, and ultimately maternal and newborn healthcare outcomes. Several factors at different levels could be barriers to postnatal care guideline implementation such as the inadequate adaptation of guidelines, limited incorporation of women-centred approaches, and insufficient prioritization of PNC within health systems, policy frameworks, and financial allocations [39].

Proxies of known risk factors for women such as age, parity, multiple births, previous termination, had no significant association with early discharge in our findings. Similar findings were noted in Guinea [11], suggesting that timing of discharge is mostly determined by organizational decisions rather than to meet women’s needs and preferences. Additionally, factors related to women’s perception of quality of care were not significantly associated with early discharge, suggesting that women’s experience of care are not taken into consideration when deciding time of discharge. On the other hand, women whose newborns were born underweight had lower odds of being discharged early after a caesarean section. One hypothesis is that some births by caesarean section were indicated by complications such as pre-eclampsia to prevent further deterioration in the mother. In these cases, the newborn may be underweight/premature, and mother and baby stay longer to recover. However, it is not clear in DHS data if women who stay longer with the newborn continue to receive care and be clinically followed. Women who had no ANC checks had lower odds of early discharge compared to those who had four or more. This could mean that women who had more ANC checks received more content of care (e.g. in terms of counselling on breastfeeding) that could have signalled to providers that they are more ‘ready’ to leave the health facility prior to 24 h. From another perspective, the lack of ANC attendance might have signalled to healthcare providers that women could be at higher risk and require more postnatal counselling and longer monitoring.

Contrary to the literature [10, 36], our results showed that higher education level was associated with higher odds of early discharge following birth by caesarean section. Women with lower education may be more at risk of maternal morbidity [40] therefore needing to stay longer in the facility for recovery. Additionally, it is possible that women with lower education stayed longer in the facility to ensure full recovery before discharge as providers acknowledge their vulnerability and attempt to counter the possibility that they might not return for an outpatient postnatal check [41]. On the other hand, it is also possible that women with higher levels of education have more social capital and better conditions to recover at home, and are therefore more capable of negotiating earlier discharge [10].

### Implications for research and practice

Implications for future research include the need for in-depth understanding of factors underlying the incomplete adherence to recommendations leading to early discharge. This includes qualitative research and exploration of postnatal care guideline implementation in Tanzania, especially at lower-level (primary) healthcare facilities. Also of interest is exploring the content, frequency and quality of immediate postnatal care and discharge preparedness, and women and care providers’ preferences. Future research should also explore effects of length-of-stay on maternal and newborn outcomes in low-resource settings, including early detection and management of complications, and maternal and neonatal mortality.

From the practice side, our study highlights the need to ensure that the conditions needed for guideline implementation, including facility infrastructure for enabling positive immediate postnatal period, are consistently available in all levels of healthcare facilities and across regions to reduce inequalities in care provision and access to quality care among all women. There is a need to integrate person-centred care in recommendations and practice, to involve women and families in decisions of postpartum discharge by adopting participatory approaches that take into consideration available resources, care providers’ needs, and women’s and families’ choices and preferences.

### Strengths and limitations

This study presents nationally representative estimates and periodic change in early discharge in Tanzania, and informs recommendations to improve immediate postnatal care in Tanzania. Nonetheless, some limitations of the work include the absence of information on maternal and newborn complications during pregnancy, intrapartum and postpartum which are important determinants of length-of-stay [42, 43]. The analysis also allowed to explore issues with data quality related to specific variables in the Tanzanian DHS, especially those related to respectful quality care which at one point signal overreporting of good quality of care leading us to drop some variables from the analysis (e.g. birth companionship prevalence was ~ 80% in 2022 DHS data while the literature shows that this value is overestimated [44]). Other reporting bias including recall bias are possible to affect the data quality – for example a proportion of women who had a caesarean section reported having it in a dispensary which is a level of facility that is not usually equipped to provide caesarean sections. Excluding women who stayed longer than 3 weeks as outliers could exclude some women who had severely premature newborns or other complications, however we used this cut-off consistently with previous research on the topic [10].

## Conclusion

Despite reduction of short postpartum stays after vaginal births, early postpartum discharge affected a third of the women giving birth in Tanzania. Early discharge could be introducing a preventable risk of missing the identification and treatment of complications, considering that around one in five women who give birth in a health facility in Tanzania reported not receiving a postnatal check [41]. Inequalities and inconsistencies in guideline application were identified in the study, warranting the need to ensure equitable provision of adequate quality of postnatal care in all geographic zones and levels of healthcare facilities. More attention to improving immediate and longer-term postnatal care is recommended at the national level.

## Supplementary Information

Below is the link to the electronic supplementary material.

Supplementary Material 1

## Data Availability

This paper uses secondary data that were made available by the DHS program at the time that the database was acquired. A data request could be made to the DHS program through this link: https://dhsprogram.com/data/dataset/Tanzania_Standard-DHS_2022.cfm?flag=0. Authors do not hold ownership of the data and this reason prevents us from sharing the data with the manuscript.
