# Supplementary material for: Discharge time from healthcare facilities after birth in Tanzania : a secondary analysis of demographic and health surveys from 2015 to 16 and 2022
Source: BMC Health Serv Res. 2026 Jan 14;26:212. doi: 10.1186/s12913-026-14035-x (PMC12888493; doi:10.1186/s12913-026-14035-x)

Table S1 – Definition of variables included in the study

Outcome / indicator	Question code	Variable code in individual recode dataset	Variable used in analysis
Length-of-stay (LoS) after most-recent facility-based livebirth	Q431	m61_1	Continuous – converted to hours based on Campbell et al 2016
Early discharge – cut-offs according to WHO and Tanzania Ministry of Health	Q431	m61_1	Categorical – 2 categories<br>Coded from continuous LoS variable:<br>1= Early discharge (Yes) when LoS<24 hours after vaginal birth or LoS<72 hours after caesarean section<br><br>0 = Recommended LoS (No) when LoS≥24 hours after vaginal birth or LoS≥72 hours after caesarean section

Variable	Question code	Variable code in IR dataset	Variable as used in analysis	DHS 2015-16	DHS 2022
Zone		sreg1	Categorical – 9 categories<br>Western, Northern, Central, Southern highlands, Southern, South west highlands, Lake, Eastern and Zanzibar	x	x
Residence		v025	Categorical – 2 categories<br>0= Rural<br>1= Urban	x	x
Union and cohabiting status (at time of survey)	Q701, Q704, Q703, Q704	v502 and v504	Categorical – 2 categories<br>0= Not in union/not living with partner<br>1= In union and living with partner	x	x
Facility level	Q430	m15_1	Categorical – 3 categories<br>0= Hospital<br>1= Health centre<br>2= Dispensary/clinic	x	x
Facility ownership	Q430	m15_1	Categorical – 2 categories<br>0= Governmental<br>1= Non-governmental	x	x
Day of birth	Q215	b17_01, b1_01, b2_01	Categorical – 2 categories<br>0= Weekend (Saturday, Sunday)<br>1= Weekday (Monday-Friday)<br>Days of the week generated from day of birth using '*dow'* function in Stata.	x	x
Shared bed during stay	Q446A.a	mh18a_1	Categorical – 2 categories<br>0 = No<br>1= Yes		x
Spent time without a mattress on the floor	Q446A.b	mh18b_1	Categorical – 2 categories<br>0 = No<br>1= Yes		x
Was denied care because could not pay	Q446B	mh19_1	Categorical – 2 categories<br>0 = No<br>1= Yes		x
Was delayed in leaving the facility because could not pay	Q446C	mh20_1	Categorical – 2 categories<br>0 = No<br>1= Yes		x
Access to toilet	Q446F-G	mh23_1 & mh24_1	Categorical – 2 categories<br>0 = No<br>1= Yes		x
Maternal age at index childbirth [newborn's date of birth – respondent's date of birth]		b3_01 – v011	Categorical – 5 categories<br>0= <19 years<br>1= 20-24 years<br>2= 25-29 years<br>3= 30-34 years<br>4= 35+ years	x	x
Highest completed education level	Q107-Q108	v106	Categorical – 3 categories<br>1= No formal education<br>2= Primary education<br>3= Secondary education or higher	x	x
Household wealth index for urban / rural		v190a	Categorical – 5 categories<br>0= Poorest ; 1= Poorer<br>2= Middle ; 3= Richer ; 4= Richest	x	x
Parity at index birth	Birth history	b0_01-b0_20	Categorical – 3 categories – # pregnancies (multiple pregnancies counted as 1)<br>1= Primiparous<br>2= Multiparous 2-4<br>3= Grand multiparous 5+	x	x

Variable	Question code	Variable code in IR dataset	Variable as used in analysis	DHS 2015-16	DHS 2022
Number of ANC visits during index pregnancy	Q408<br>Q412	m14_1	<u>Categorical – 3 categories</u><br>0= None<br>1= 1-3 visits<br>2= 4 or more visits	x	x
Mode of birth	Q432	m17_1	<u>Categorical – 2 categories</u><br>0= Vaginal birth<br>1= Birth by caesarean section	x	x
Multiple births	Q214	b0_01	<u>Categorical – 2 categories</u><br>0= Single birth<br>1= Multiple birth	x	x
Ever had a terminated pregnancy	Q206 /<br>Q230	v228	<u>Categorical – 2 categories</u><br>0= Never had a terminated pregnancy<br>1= Ever had a terminated pregnancy	x	x
Newborn sex	Q213	b4_01	<u>Categorical – 2 categories</u><br>0= Boy<br>1= Girl	x	x
Newborn underweight status at birth	Q428	m19_1	<u>Categorical – 2 categories</u><br>0 = not underweight (weight >=2500g)<br>1= underweight (weight < 2500)<br><br>n=247 missing values	x	x
Newborn survival and time of death		b5_01, b6_01, m61_1	<u>Categorical – 3 categories</u><br>0= Survived until survey<br>1= Died on/before day of discharge<br>2= Died after discharge<br><br>Recoded following the guidance in Campbell et al 2016.	x	x

Table S2 – Characteristics of women (15-49 years) who had their most recent livebirth in a healthcare facility with missing/unknown values for length-of-stay in the three years preceding the DHS survey.

	Characteristics	2015-16 (n=3)	2022 (n=36)
Socio-demographic and economic factors	**Zone**		
	Western	0	1 (3.7%)
	Northern	0	1 (2.4%)
	Central	0	2 (6.4%)
	South west highlands	1 (22.7%)	10 (16.3%)
	Lake	1 (66.5%)	19 (51.9%)
	Eastern	0	3 (19.2%)
	Zanzibar	1 (10.8%)	0
	**Residence**		
	Rural	3 (100%)	27 (72%)
	Urban	0	9 (28%)
	**Union and cohabiting status at time of survey**		
	Not in union/not living with a partner	1 (66.5%)	6 (12.9%)
	Living with a partner	2 (33.5%)	30 (87.1%)
	**Maternal age at index childbirth**		
	13-19 years	2 (89.2%)	4 (7.0%)
	20-24 years	0	11 (28.1%)
	25-29 years	1 (10.8%)	9 (24.4%)
	30-34 years	0	6 (24.6%)
	35-49 years	0	6 (15.8%)
	**Highest completed education level**		
	No formal education	2 (89.2%)	13 (31.3%)
	Primary education	1 (10.8%)	15 (42.9%)
	Secondary or higher	0	8 (25.8%)
	**Household wealth index**		
	Poorest	0	15 (37.8%)
	Poorer	1 (22.7%)	12 (24.4%)
	Middle	0	3 (12.1%)
	Richer	1 (66.5%)	3 (10.3%)
	Richest	1 (10.8%)	3 (15.3%)
Facility characteristics and perception of care quality	**Facility level**		
	Dispensary/clinic	1 (22.7%)	17 (41.6%)
	Health center	0	11 (29.9%)
	Hospital	2 (77.3%)	8 (28.5%)
	**Facility ownership**		
	Governmental	3 (100%)	36 (100%)
	**Day of birth**		
	Weekend	0	12 (33.2%)
	Weekday	3 (100%)	24 (66.8%)
	**Shared bed during stay**	N/A	1 (2.4%)
	**Spent time without a mattress on the floor**	N/A	1 (2.8%)
	**Was denied care because could not pay**	N/A	0
	**Was delayed to leave the facility because could not pay**	N/A	0
	**Access to toilet**	N/A	30 (86.9%)
Women's needs and obstetric history	**Mode of birth**		
	Vaginal	3 (100%)	33 (91.2%)
	Caesarean section	0	3 (8.8%)
	**Parity at index birth**		
	Primiparous	3 (100%)	9 (21%)
	Multiparous 2-4	0	17 (49.3%)
	Multiparous 5+	0	10 (29.7%)
	**Number of ANC visits during index pregnancy**		
	None	0	21 (56.3%)
	1-3 visits	2 (89.2%)	3 (8.2%)
	4 or more visits	1 (10.8%)	12 (35.4%)
	**Multiple birth**	3 (100%)	0
	**Ever had a terminated pregnancy**	2 (33.5%)	0
Newborn characteristics	**Newborn sex**		
	Boy	3 (100%)	17 (48.8%)
	Girl	0	19 (51.2%)
	**Newborn underweight status at birth**[β]		
	Underweight <2500g	0	1 (3%)
	Not underweight ≥2500g	3 (100%)	28 (97%)
	**Newborn survival**		
	Survived until survey	3 (100%)	36 (100%)
	**Total**	3 (100%)	36 (100%)

[β]n=7 missing values for newborn's weight at birth in 2022

Table S3 - **Multivariable logistic regression of factors and early discharge among women who had their most recent birth (vaginal and cesarean section) in a healthcare facility in the three years preceding the DHS 2022, excluding variables with missing values**

	Characteristics	Vaginal births (n=3,994) aOR (95%CI)	Vaginal births p-value	Cesarean section (n=624) aOR (95%CI)	Cesarean section p-value
Socio-demographic and economic factors	**Zone†**				
	Western	0.6 [0.4;1.0]	0.065	0.8 [0.3;2.6]	0.749
	Northern	1.2 [0.8;1.9]	0.423	0.7 [0.3;1.5]	0.357
	Central	0.8 [0.5;1.2]	0.288	1.4 [0.6;3.5]	0.470
	Southern highlands	0.5 [0.3;0.9]	0.014	0.5 [0.2;1.1]	0.071
	Southern	0.9 [0.6;1.6]	0.825	0.8 [0.2;2.5]	0.667
	South west highlands	1.9 [1.3;2.9]	0.002	0.8 [0.3;1.8]	0.568
	Lake	0.7 [0.5;1.0]	0.080	1.3 [0.5;3.3]	0.560
	Eastern	ref		ref	
	Zanzibar	6.4 [4.1;9.9]	<0.001	1.6 [0.6;4.5]	0.330
	**Residence**				
	Rural	ref		ref	
	Urban	1.0 [0.8;1.3]	0.934	1.6 [1.0;2.6]	0.070
	**Union and cohabiting status at time of survey**				
	Not in union/not living with a partner	ref			
	Living with a partner	1.1 [0.9;1.4]	0.211		
	**Maternal age at index childbirth**				
	13-19 years	0.9 [0.7;1.4]	0.770		
	20-24 years	ref			
	25-29 years	1.1 [0.8;1.4]	0.687		
	30-34 years	1.2 [0.9;1.6]	0.301		
	35-49 years	1.1 [0.8;1.6]	0.600		
	**Highest completed education level^**				
	No formal education	1.0 [0.7;1.2]	0.771	0.3 [0.1;0.8]	0.023
	Primary education	ref		ref	
	Secondary or higher	0.9 [0.7;1.2]	0.596	1.0 [0.6;1.7]	0.901
	**Household wealth index**				
	Poorest	1.0 [0.8;1.4]	0.846	0.4 [0.1;1.2]	0.114
	Poorer	0.8 [0.6;1.1]	0.156	1.0 [0.4;2.1]	0.946
	Middle	ref		ref	
	Richer	0.9 [0.7;1.2]	0.406	1.3 [0.7;2.6]	0.389
	Richest	0.8 [0.6;1.2]	0.364	1.3 [0.6;2.7]	0.478
Facility characteristics and perception of	**Facility level†**				
	Dispensary/clinic	3.6 [2.6;5.0]	<0.001	--	--
	Health center	1.5 [1.1;2.0]	0.008	3.2 [2.0;5.4]	<0.001
	Hospital	ref		ref	
	**Facility Ownership**				
	Governmental	ref		ref	
	Non-governmental	0.8 [0.5;1.3]	0.394	0.7 [0.4;1.4]	0.303
Women's needs and obstetric history	**Parity at index birth**				
	Primiparous	ref			
	Multiparous 2-4	1.0 [0.7;1.3]	0.769		
	Multiparous 5+	1.1 [0.7;1.5]	0.706		
	**Number of ANC visits during index pregnancy†**				
	None	0.7 [0.5;1.0]	0.041		
	1-3 visits	1.0 [0.8;1.2]	0.932		
	4 or more visits	ref			
Newborn characteristics	**Newborn survival**				
	Survived until survey	ref			
	Died on/before discharge	0.6 [0.2;1.7]	0.302		
	Died after discharge	0.6 [0.3;1.6]	0.318		

Table S4 – Length of stay and early discharge by mode of birth and survey year

		Length-of-stay (hours)		Early discharge	
		Median	IQR	n (%)	95%CI
2015-16	Vaginal birth	16	[6;36]	1890 (54.8%)	[52.4; 57.2]
	Birth by Caesarean section	84	[84;204]	47 (17.2%)	[11.3; 25.3]
	Total	24	[8;36]	1937 (51%)	[48.7; 53.3]
2022	Vaginal birth	24	[12;36]	1386 (30.3%)	[28.2; 32.5]
	Birth by Caesarean section	84	[60;108]	161 (25.2%)	[21.3; 29.5]
	Total	36	[16;60]	1547 (29.6%)	[27.7; 31.6]

Figure S1 - Histograms of length of stay distribution by mode of birth for women who had a livebirth in the three years preceding the survey in Tanzania in 2015-16 and 2022

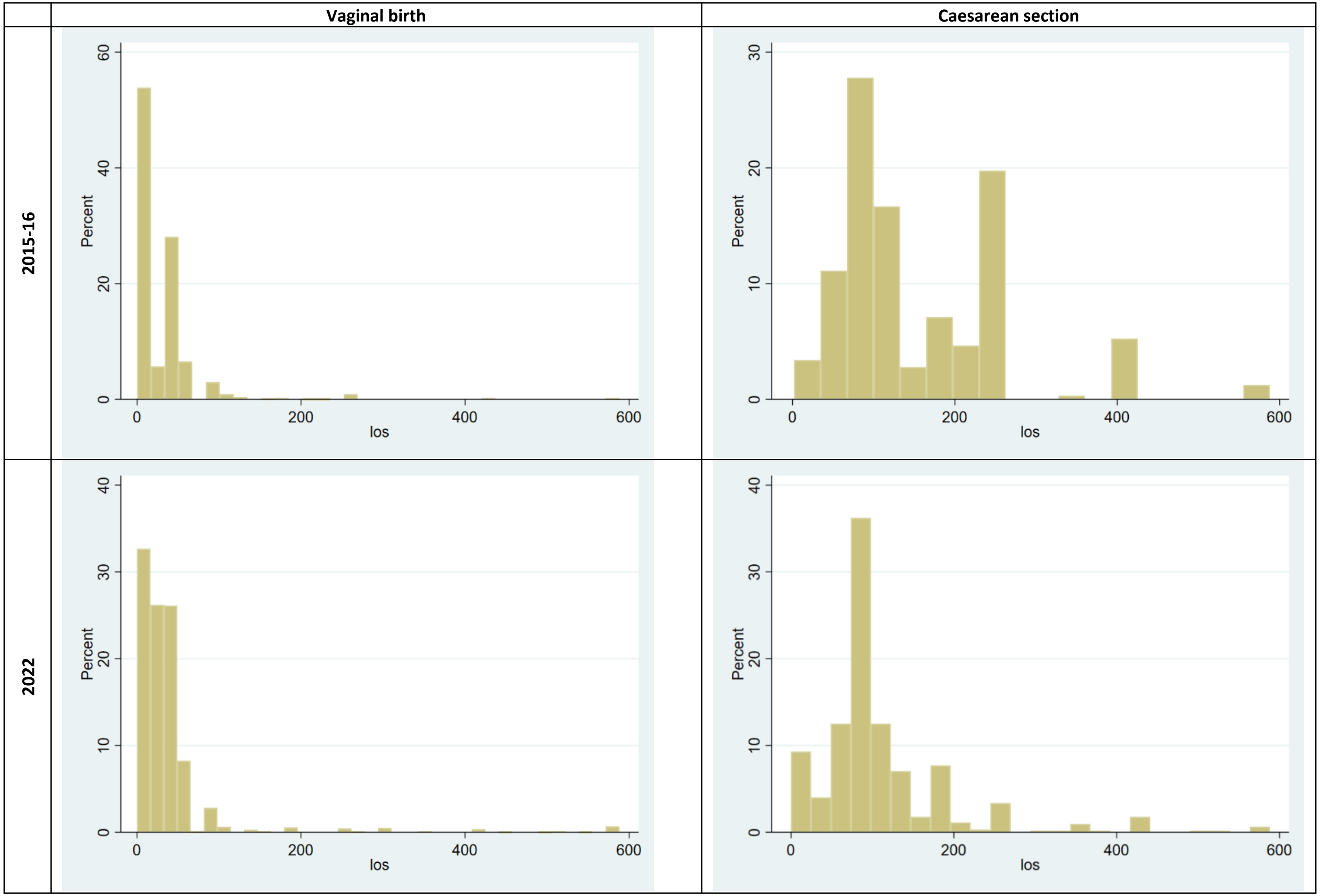

Supplement: Supplementary file 1 — Supplementary Material 1 [file 12913_2026_14035_MOESM1_ESM.pdf]
